# Loss of Calponin 2 causes age‐progressive proteinuria in mice

**DOI:** 10.14814/phy2.15370

**Published:** 2022-09-18

**Authors:** Tzu‐Bou Hsieh, Jian‐Ping Jin

**Affiliations:** ^1^ Department of Obstetrics & Gynecology Wayne State University School of Medicine Detroit Michigan USA; ^2^ Department of Physiology Wayne State University School of Medicine Detroit Michigan USA; ^3^ Department of Physiology and Biophysics University of Illinois at Chicago College of Medicine Chicago Illinois USA

**Keywords:** calponin 2, cytoskeleton, podocytes, proteinuria

## Abstract

Proteinuria is a major manifestation of kidney disease, reflecting injuries of glomerular podocytes. Actin cytoskeleton plays a pivotal role in stabilizing the foot processes of podocytes against the hydrostatic pressure of filtration. Calponin is an actin associated protein that regulates mechanical tension‐related cytoskeleton functions and its role in podocytes has not been established. Here we studied the kidney phenotypes of calponin isoform 2 knockout (KO) mice. Urine samples were examined to quantify the ratio of albumin and creatinine. Kidney tissue samples were collected for histology and ultrastructural studies. A mouse podocyte cell line (E11) was used to study the expression and cellular localization of calponin 2. In comparison with wild‐type (WT) controls, calponin 2 KO mice showed age‐progressive high proteinuria and degeneration of renal glomeruli. High levels of calponin 2 are expressed in E11 podocytes and colocalized with actin stress fibers, tropomyosin and myosin IIA. Electron microscopy showed that aging calponin 2 KO mice had effacement of the podocyte foot processes and increased thickness of the glomerular basement membrane as compared to that of WT control. The findings demonstrate that deletion of calponin 2 aggravates age‐progressive degeneration of the glomerular structure and function as filtration barrier. The critical role of calponin 2 in podocytes suggests a molecular target for understanding the pathogenesis of proteinuria and therapeutic development.

## Graphical abstract

Calponin 2 KO mice develop age‐progressive proteinuria. The data show significantly higher urine albumin/creatinine ratio in 18‐24 months old calponin 2 KO than that in age‐matched WT mice. (a) compared to 3‐8 months old WT group; (b) compared to age‐matched WT and 3‐8 months old KO groups.

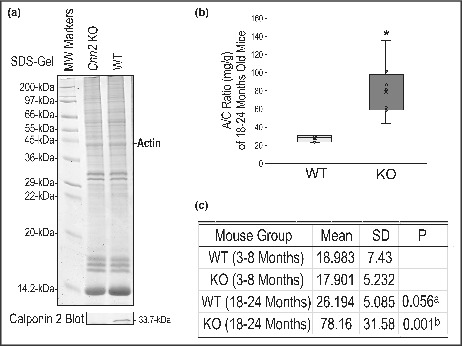

## 1 INTRODUCTION

Chronic kidney diseases affect more than 10% of the general population globally, and most of the pathogenesis originates from glomerulus injuries (Assady et al., 2019). There is a steady decrease in renal glomerular filtration rate by 10% per decade after age 35 (Glassock & Rule, 2016). Therefore, aging‐related decline of kidney function increases disease susceptibility and aggravates the incidence of chronic kidney diseases. The deterioration in kidney function is due mostly to the loss of functional nephrons from podocyte depletion, glomerulosclerosis, tubular atrophy, and interstitial fibrosis (Zhang et al., 2019). Regardless of the initial cause of glomerular injury, proteinuria is usually the most common and major manifestation of kidney diseases. While proteinuria may occur transiently in healthy subjects after strenuous exercise or during pregnancy (Weissgerber et al., 2014), persistent and progressive proteinuria usually leads to end‐stage kidney failure.

Renal filtration occurs in the glomerulus that has a barrier structure consisting of three layers: fenestrated capillary endothelium, the glomerular basement membrane (GBM), and the podocytes. Podocytes are highly differentiated epithelial cells that cover the outer layer of the glomerular tufts with projected primary and secondary foot processes. The primary foot processes (PFP) attach directly to the GBM through adhesion molecules and interdigitate with neighboring PFPs bridged with slit diaphragm (SD) to provide a major support to sustain the integrity of glomerular capillary against high filtration pressure (Endlich & Endlich, 2006). The SD is composed of two groups of proteins: Podocyte‐specific proteins, such as nephrin, nephrin‐like‐1, and podocin, as well as cell junctional proteins, such as P‐cadherin, zonula occluden‐1, and FAT, to connect adjacent PFP and allow the passage of fluid while preventing the loss of protein and other macromolecules (Martin & Jones, 2018). Studies have shown that specialized proteins, such as podocin and CD2‐associated protein, are crucial for anchoring SD to actin microfilaments in podocytes and maintaining the functional cellular architecture (Leeuwis et al., 2010). Therefore, factors affecting the stability and functional integrity of actin cytoskeleton may lead to podocyte injury and kidney diseases.

Calponin is an actin filament‐associated protein and regulates cytoskeleton functions via inhibiting actin‐activated myosin ATPase and motor activities (Abe et al., 1990; Haeberle, 1994; Winder & Walsh, 1990). Three isoforms of calponin, calponin 1, calponin 2, and calponin 3, have evolved in vertebrates as the products of homologous genes, *Cnn1, Cnn2, and Cnn3,* respectively (Jin et al., 2008; Liu & Jin, 2016). Calponin 1 is specifically expressed in differentiated smooth muscle cells and is the most studied isoform for its function in the regulation of smooth muscle contractility (Feng et al., 2019; Jin et al., 2008; Nigam et al., 1998). Calponin 3 is expressed in smooth muscle cells (Applegate et al., 1994), brain (Trabelsi‐Terzidis et al., 1995), trophoblasts (Shibukawa et al., 2010), and myeloid cells (Flemming et al., 2015; Shibukawa et al., 2013). Calponin 2 is expressed in smooth muscles (Hossain et al., 2003) and multiple types of non‐muscle cells, such as epithelial cells (Hossain et al., 2006), fibroblasts (Hossain et al., 2005), endothelial cells (Tang et al., 2006), and macrophages (Huang et al., 2008).

Previous studies on calponin 2 have established its role in maintaining a physiological level of mechanical equilibrium and the stability of actin cytoskeleton (Jensen et al., 2014). By adjusting the tension and stability of actin filaments through inhibitory regulation of myosin II‐dependent cell traction force (Hossain et al., 2016), calponin 2 regulates actin‐cytoskeleton‐based cellular functions, such as migration (Moazzem Hossain et al., 2014), phagocytosis (Huang et al., 2008), and proliferation (Hossain et al., 2003). Calponin 2 knockout (KO) mice exhibit less inflammatory response to surgical injury (Hsieh et al., 2021) and develop milder inflammatory arthritis (Huang et al., 2008), indicating its role in macrophage and fibroblast functions.

The gene expression and protein stability of calponin 2 are both regulated by mechanical tension (Hossain et al., 2005;2006). The average tension over time, other than dynamic tension, determines the expression of calponin 2 as shown with lung alveolar cells (Hossain et al., 2006). The levels of calponin 2 vary in different cell types, implicating a positive relationship to the level of native environmental mechanical tension to which the cells have adapted (Hossain et al., 2006). A previous study reported the presence of calponin in podocytes without determining the isoform (Saleem et al., 2008). For actin cytoskeleton's role in podocytes and their intercellular adhesion under high hydrostatic pressure and tension (Endlich & Endlich, 2006), we hypothesize that calponin 2 may play a role in sustaining the structure and function of podocytes and their foot processes. To investigate this hypothesis, here we studied calponin 2 KO mice and a podocyte cell line as model systems to demonstrate that podocytes express high levels of calponin 2 and deletion of calponin 2 resulted in age‐progressive proteinuria. Structural and functional evidence suggests a novel molecular target for understanding the pathogenesis of proteinuria and therapeutic development.

## 2 MATERIALS AND METHODS

### 2.1 Genetically modified mice

All animal procedures were performed using protocols approved by the Institutional Animal Care and Use Committee of Wayne State University.

Mice were maintained in a temperature and humidity‐controlled room with a 12‐h light/dark cycle and free access to food and water. Calponin 2 KO (*Cnn2* ^*−/−*^) mice were developed and bred in the house as described previously (Huang et al., 2008). Briefly, two tandem *loxP* sequencies were inserted into intron 1 and intron 2 of the *Cnn2* gene to allow Cre recombinase‐catalyzed deletion of exon 2. After transfection and cloning of embryonic stem cells, *Cnn2*‐targeted chimeric mice were generated in C57BL/6 strain. The *Cnn2‐flox* mice were crossed with *Zp3‐Cre* mice to generate systemic knockout of *Cnn2* gene expression in the offspring. The colony of calponin 2 KO mice has been backcrossed with C57BL/6 mice from the Jackson Lab for more than 10 generations to ensure the genetic background. Genotyping was done using PCR on tail biopsies and confirmed in all experimental mice post‐mortem by Western blot analysis of protein extracts from the spleen (Huang et al., 2016). Tweny two pairs of age‐matched calponin 2 KO (*Cnn2* ^*−/−*^) and wild type (WT) littermate (*Cnn2* ^*+/+*^) of both sexes were studied in this project. The age of the mice studied ranged from 3 to 24 months old.

### 2.2 Anti‐calponin 2 antibodies used in the study

A polyclonal antibody (RAH2) was raised in rabbit against purified mouse calponin 2 antigen as described previously (Nigam et al., 1998). A monoclonal antibody (mAb) 1D11 specific to calponin 2 was developed by immunization using mouse calponin 2 antigen as described previously (Hossain et al., 2006). The specificity of these antibodies has been verified using Western blot analysis against purified calponin 1 and 2 proteins. RAH2 reacted to calponin 2 with a weak cross‐reaction to calponin 1 and 1D11 reacted specifically to calponin 2 (Feng et al., 2019).

### 2.3 SDS‐PAGE and Western blotting analysis

SDS‐polyacrylamide gel electrophoresis (PAGE) and Western blotting were carried out as described previously (Huang et al., 2008). Briefly, purified protein, tissue homogenate, and cell lysate in SDS‐gel sample buffer (50 mM Tris–HCl, pH 8.8, 2% SDS, 140 mM β‐mercaptoethanol, 0.1% bromophenol blue, 10% glycerol) were heated at 80°C for 5 minutes (the lysates of cultured cells were treated by passing through a 25G needle for more than 20 times to shear the chromosomal DNA and reduce viscosity), and clarified by centrifugation in a microcentrifuge at 20,000 × g for 5 min. The protein samples were analyzed on 12% SDS‐gel with acrylamide to bis‐acrylamide ratio of 29:1 in the Laemmli discontinuous buffer system. Resolved gels were stained with Coomassie Blue R‐250 to reveal the protein bands.

Duplicate gels were transferred onto nitrocellulose membranes using a Bio‐Rad semidry transfer apparatus for Western blot analysis. The blotted membranes were blocked with 1% bovine serum albumin (BSA) in Tris‐buffered saline (TBS, 150 mM NaCl and 50 mM Tris–HCl, pH 7.5) at room temperature for 30 min, then incubated with polyclonal antiserum RAH2 (Nigam et al., 1998) at 1:2000 dilution in TBS containing 0.1% BSA at room temperature for 1 h. After three washes with TBS containing 0.05% Tween‐20 for 7 min each and three washes with TBS for 3 min each, the membranes were incubated with alkaline phosphatase‐labeled anti‐rabbit IgG secondary antibody (Sigma, A2306) at 1:5000 dilution in TBS containing 0.1% BSA at room temperature for 1 h. Washed again as above, the membranes were developed in 5‐bromo‐4‐chloro‐3‐indolyl phosphate/nitro blue tetrazolium chromogenic substrate solution to reveal the calponin 2 band (Hossain et al., 2003).

Densitometry scans of the actin bands on SDS gels and the calponin 2 bands on Western blots were performed using an Epson Perfection Scanner (V800) at 600 dots per inch and analyzed using NIH ImageJ software version 1.61. Quantification of the relative level of calponin 2 expression was carried out by normalizing to the actin band in parallel SDS‐gel. This method has shown reproducible reliability in previous studies of calponin 2 expression in various cell types or tissues (Hossain et al., 2006; Huang et al., 2008; Sato et al., 1975).

### 2.4 Collection of urine and kidney samples

Clean catch spot urine samples without fecal contamination were collected from the mice in the morning between 8:00 and 10:00 a.m. The urine samples were stored at −80°C before quantification for albumin and creatinine. No manipulation or compression on the abdomen of the mouse was applied during collection to avoid contamination of the urine samples.

At designated age, the mice were euthanized under deep anesthesia using isoflurane followed by cervical dislocation and the body weight was recorded. After laparotomy, the kidneys were excised, carefully trimmed to remove the perinephric fat and vessels, recorded for weight, and processed for histological and ultrastructure studies.

### 2.5 Quantification of urine albumin and creatinine

Urine albumin was measured using a BCP Albumin Assay Kit (Sigma, MAK125) and urine creatinine was quantified using a Creatinine Assay Kit (Sigma, MAK080) according to the manufacturers' protocols. Urine albumin excretion rate was determined from the albumin to creatinine ratio.

### 2.6 Histology and histochemistry

The entire kidney was fixed in 4% paraformaldehyde in phosphate‐buffered saline (PBS, pH 7.4) overnight and proceeded for paraffin embedding using standard methods. Four μm serial sections were cut and stained with Mayer's hematoxylin and eosin Y (Electron Microscopy Scientific, 87,019) or periodic acid‐Schiff staining (Sigma, 395B‐1KT) following the manufacturers' instructions for histology studies. The slides were examined using a Keyence epifluorescence microscope (BZX810) with analysis software.

### 2.7 Quantitative measurements of renal histopathology

**FIGURE 1:**
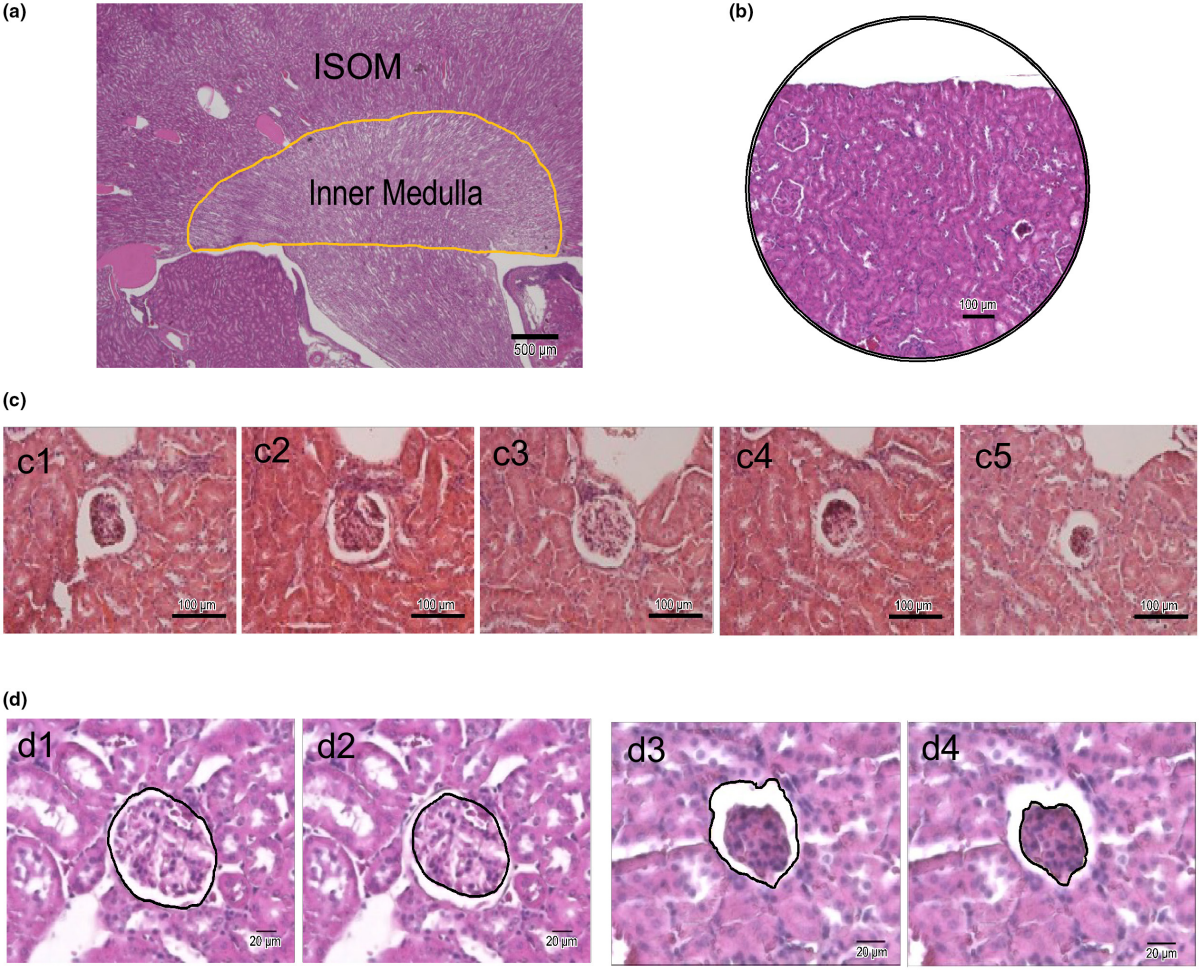
Approaches to quantify histological images of kidney sections. (a) a representative image of a PAS stained 8 μm thick mid‐sagittal kidney section from an 18 months old calponin 2 KO mouse. The renal portion of the inner medulla was outlined with the yellow line indicating the area used for quantification and comparison between different groups of mice. (b) To standardize the area for counting glomerular numbers, the edge of the renal cortex was placed at the upper 1/3 of the visual field for calculating the glomerular counts and G/B ratio. H&E stain was used. (c) Representative images of five H&E stained serial sections of the kidney of a WT mouse demonstrate the selection of the largest diameter of the glomerulus for calculating G/B ratio. (c3) displayed the largest diameter compared to the adjacent two sections (c2 & c4) and was selected for calculating G/B ratios for comparison. (d) On H&E stained images, the area of Bowman's capsule was outlined from the inner surface of the Bowman's capsule and the area of glomerular tuft was measured from the outer surface of the glomerular capillary. Represented photos of a glomerulus from an 18 months old WT mice with a glomerular G/B ratio of 73.2% (d1 & d2) and from an 18 months old calponin KO mice with a glomerular G/B ratio of 54.5% were shown (d3 & d4).

High amounts of albumin leaked from glomerular injury would accumulate in the renal tubules and aggregates to form protein casts. Therefore, the number of dilated renal tubules with proteinaceous casts was counted in mid‐sagittal sections of the kidney stained with periodic acid‐Schiff reagent under a light microscope to demonstrate protein leaked from the glomerulus and accumulated in the renal tubule (Dvanajscak et al., 2020; Nolin et al., 2016). Our approach was to take measurements in the medulla for the more visible casts in larger collecting tubules. The entire inner stripe of the outer medulla was inspected to count the number of dilated renal tubules that contain protein casts. Dilated tubules were defined as the diameter larger than twice of the diameter of the adjacent normal tubules. Sections with the widest renal medulla were selected for calculating the number of dilated renal tubule of each mouse. Since all renal tubules course from the outer medulla to the inner medulla and end in collecting tubules, the area of inner medulla can be used as a reference of the relative number of renal tubules. To compare the number of dilated renal tubules between calponin 2 KO and WT groups, the number of dilated renal tubules was normalized to the area of renal portion of the inner medulla (Figure 1a).

Total glomerular count was examined on kidney sections cut from the midsagittal plan to include the largest cortical portion. The renal cortex was divided into three areas, namely upper pole, midzone, and lower pole. Four nonoverlapping areas were chosen from each zone for quantification. Under 100× magnification, the edge of the renal cortex was placed at the upper 1/3 of the visual field for counting glomerular number. All glomeruli within the renal cortex were included and counted (Figure 1b). Total glomerular counts were obtained as the sum of glomerular number in the 12 areas.

The percentage of glomeruli with low glomerular tuft to Bowman's capsule area ratio (G/B ratio) was quantified. The reported mean diameter of the mouse glomerulus is around 80 μm (Sato et al., 1975), therefore, five serial sections of 8 μm thickness should include a section with the largest diameter of each glomerulus for the measurements (Figure 1c). The area of glomerular tuft was measured from the outer surface of the glomerular capillary and the area of Bowman's capsule was outlined from the inner surface of the Bowman's capsule (Figure 1d) using ImageJ software and converted into mm^2^. Glomeruli with low G/B ratio were defined as G/B ratio <60%. The percent low G/B ratio glomeruli of total glomeruli was determined for each mouse.

### 2.8 Podocyte cultures

A conditionally immortalized murine podocyte cell line E11 (Schiwek et al., 2004) (passage 33) was purchased from Cell Line Services (Eppelheim, Germany). The E11 podocytes were cultured in PRMI 1640 (Global Life) supplemented with 10% fetal bovine serum, penicillin, streptomycin, and glutamine in 5% CO_2_ atmosphere at 33°C to reach ~60% of confluence. Cells were expanded for storage in liquid nitrogen or direct use in experiments. Cells from passages 35 and 36 were used in the present study. Differentiation of the podocytes was induced by continuing the culture at 38°C for 14 days. The cultural media were changed every 3–4 days.

After three washes with warm PBS, cell lysates were harvested from the monolayer cultures by solubilization in SDS‐gel sample buffer. In parallel cultures, cells were seeded on collagen coated cover slips (Fisher Scientific) for immunofluorescence studies. SDS‐gel samples and coverslips of proliferating and differentiated podocytes were collected at designated time points for SDS‐PAGE/Western blotting (Hossain et al., 2003; Hossain et al., 2005) and immunofluorescent studies.

### 2.9 Immunofluorescence microscopy

Kidneys were embedded in Tissue‐Tek optimal cutting temperature (O.C.T.) compound (Sakura Finetek USA) in a cryomold, rapidly frozen in liquid nitrogen, and stored at −80°C before sectioning. Serial fresh frozen sections of 8 μm thickness were cut using a ThermoFisher HM550 cryostat. Sections were collected on Fisher Superfrost Plus slides and stored at −80°C.

For immunofluorescent staining, frozen sections of the mouse kidney were air dried, fixed in 75% acetone‐25% ethanol for 10 min, and washed three times with PBS. The slides were then blocked with 3% BSA in PBS for 30 minutes and incubated with anti‐calponin 2 RAH2 antiserum at 1:400 dilution and 1D11 mouse mAb supernatant at 1:50 dilution, a rabbit anti‐podocin antibody (Sigma P0372) at 1:200 dilution, a rabbit anti‐nephrin antibody (Sigma PRS2265) at 1:200 dilution, a mouse anti‐tropomyosin mAb CG3 at 1:200 dilution (Lin et al., 1988), or a rabbit anti‐myosin IIA antibody (Abcam ab24762) at 1:200 dilution, in PBS containing 0.05% Tween‐20 (PBS‐T) and 0.1% BSA in a humidified chamber at room temperature for 1 h. After washing three times with PBS‐T for 5 min each, the coverslips and tissue sections were incubated with fluorescein isothiocyanate‐conjugated goat anti‐mouse IgG (Sigma, F1010), tetramethyl rhodamine isothiocyanate (TRITC)‐conjugated anti‐mouse IgG (Sigma, T‐5393), or FITC‐conjugated goat anti‐rabbit IgG (Sigma, F7512) secondary antibody (at 1:200 dilutions) in PBS‐T containing 0.1% BSA in a dark box at room temperature for 1 h. Washed three times for 5 min each as above, the coverslips and sections were mounted with ProLong Gold antifade reagent containing DAPI counter stain for nuclei (Life Technology) and sealed using a nail polisher. Rhodamine phalloidin (Life Technology, R37112) was used to stain the actin cytoskeleton. Tissue slides going through the same processes without adding primary antibody were used as negative control. Images were obtained using a Leica SD600 spinning disc confocal microscope and processed using image analysis software MetaMorph NX.

### 2.10 Transmission electron microscopy

Mouse kidney tissue was cut into 1 mm thick slices and fixed in 2% glutaldehyde and 2% paraformaldehyde in PBS overnight. Subsequent embedding and sectioning for transmission electron microscopy (EM) analysis were performed by the Central Microscopy Research Facility at the University of Iowa and imaged using a JEOL JEM‐1230 electron microscope.

The thickness of glomerular basement membrane (GBM) was measured as described previously (Haas, 2006; Sato et al., 2010). A total of 24 EM images at 10,000× magnification were used for the measurements. The images were viewed with standard grid overlay using ImageJ software. Two glomeruli were measured for each sample. Each glomerulus encompassed at least six complete loops of the capillary. The image of each capillary loop was divided into half from the center. Six points were randomly chosen from each side for the measurement. The thickness of GBM was measured from the lower tip of the foot process of the podocyte to the upper end of the endothelium perpendicular to the long axis of the GBM. Capillary loop with sharp angle or covered with mesangial cells were excluded. The GBM thickness was averaged to represent calponin KO and WT mice in statistical analysis.

### 2.11 Statistical analysis

IBM SPSS Statistics Version 27 was used for statistical analysis. All quantitative results are presented as means ± SD. Comparison of continuous variables between two groups were conducted by using Student's *t*‐test. *p* < 0.05 was considered statistically significant.

## 3 RESULTS

### 3.1 Calponin 2 KO mice exhibit age‐progressive proteinuria

**FIGURE 2:**
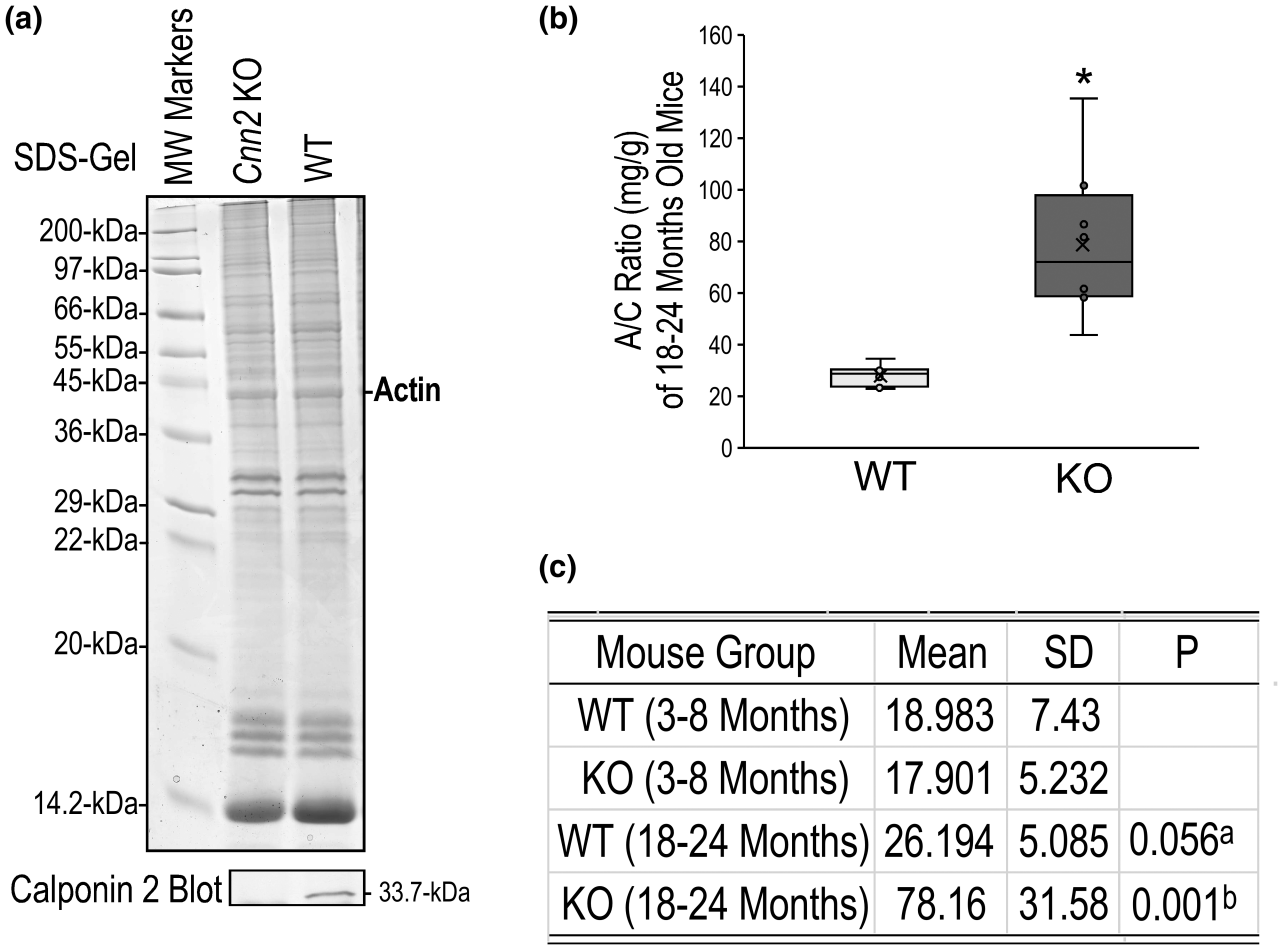
Calponin 2 KO mice develop age‐progressive proteinuria. (a) Representative SDS‐gel and Western blot using anti‐calponin 2 antibody RAH2 on spleen cell lysate verifies the loss of calponin 2 expression in KO mice and the expression of calponin 2 in WT mice. (b) The box and whisker plot comparison shows significantly higher urine A/C ratio in 18–24 months old calponin 2 KO (*n* = 8) than that in age‐matched WT (*n* = 8) mice. *p < 0.05. (c) Quantitative data summary show that no difference was found between calponin 2 KO (*n* = 7) and WT (*n* = 7) groups at 3–8 months old, demonstrating an age‐progressive development of severe proteinuria in 18–24 months old calponin 2 KO mice (*n* = 8), which was significantly more severe than that in WT control mice (*n* = 8). Values are means ± SD. Two‐tailed Student's *t*‐tests were used in the statistical tests. ^a^Compared to 3–8 months old WT group; ^b^compared to age‐matched WT and 3–8 months old calponin 2 KO groups.

The total loss of calponin 2 in the calponin 2 KO mice was confirmed in the Western blot in Figure 2a. The body weight, total kidney weight, and kidney weight/body weight ratio of the mice showed no significant differences between calponin 2 KO and WT mice in each age‐matched group (Table 1).

**TABLE 1:** No significant difference in body weight or kidney weight/body weight ratios between calponin 2 KO and WT mice. Statistical comparisons using two‐tailed Student's *t* tests showed no difference in body weight (BW) or total kidney weight/body weight ratios between calponin 2 KO and WT mice in each age‐matched group. LK, left kidney; RK, right kidney; TKW, total kidney weight. *p* Values are listed to verify the absence of statistical significance between the KO and WT groups in two‐tailed Student’s *t* test. Values are means ± SD

Age	Male group 1 (3–8 M)		Male group 2 (10–15 M)		Male group 3 (18–24 M)		Female group 1 (3–8 M)		Female group 2 (10–15 M)		Female group 3 (18–24 M)	
Strain (*n*)	WT (7)	KO (7)	WT (7)	KO (8)	WT (7)	KO (8)	WT (7)	KO (7)	WT (7)	KO (7)	WT (8)	KO (8)
BW, g	28.45 ± 1.83	30.25 ± 3.17	30.17 ± 2.42	30.74 ± 1.23	31.62 ± 2.40	31.48 ± 2.89	22.91 ± 1.87	26.37 ± 1.84	32.12 ± 2.21	26.26 ± 3.12	31.04 ± 1.92	24.42 ± 1.95
LK, mg	0.20 ± 0.03	0.22 ± 0.03	0.20 ± 0.03	0.21 ± 0.01	0.22 ± 0.03	0.22 ± 0.03	0.15 ± 0.02	0.20 ± 0.02	0.21 ± 0.02	0.19 ± 0.01	0.20 ± 0.01	0.16 ± 0.02
RK, mg	0.21 ± 0.03	0.23 ± 0.04	0.21 ± 0.03	0.21 ± 0.02	0.22 ± 0.02	0.23 ± 0.02	0.16 ± 0.03	0.19 ± 0.02	0.21 ± 0.01	0.19 ± 0.02	0.20 ± 0.01	0.17 ± 0.02
TKW, mg	0.40 ± 0.05	0.46 ± 0.07	0.41 ± 0.06	0.42 ± 0.02	0.44 ± 0.05	0.45 ± 0.05	0.32 ± 0.05	0.39 ± 0.04	0.42 ± 0.02	0.38 ± 0.03	0.41 ± 0.02	0.34 ± 0.04
TKW/BW	14.12 ± 1.38	15.10 ± 1.26	13.76 ± 1.74	13.81 ± 0.69	13.87 ± 1.04	14.25 ± 0.58	13.81 ± 1.36	15.01 ± 1.9	13.22 ± 0.94	14.51 ± 1.75	13.15 ± 0.78	13.85 ± 1.19
*p* value	0.189		0.982		0.387		0.198		0.114		0.185	

The mean values of albumin/creatinine (A/C) ratio (mg/g) of young and old calponin 2 KO and WT mice are shown in Figure 2b. No significant difference in A/C ratio was found between 3–8 months old calponin 2 KO mice and WT littermates. In contrast, 18–24 months old calponin 2 KO mice showed significantly higher A/C ratio than that of 3–8 months calponin 2 KO mice and age‐matched WT mice, demonstrating age‐related severe albuminuria.

**FIGURE 3:**
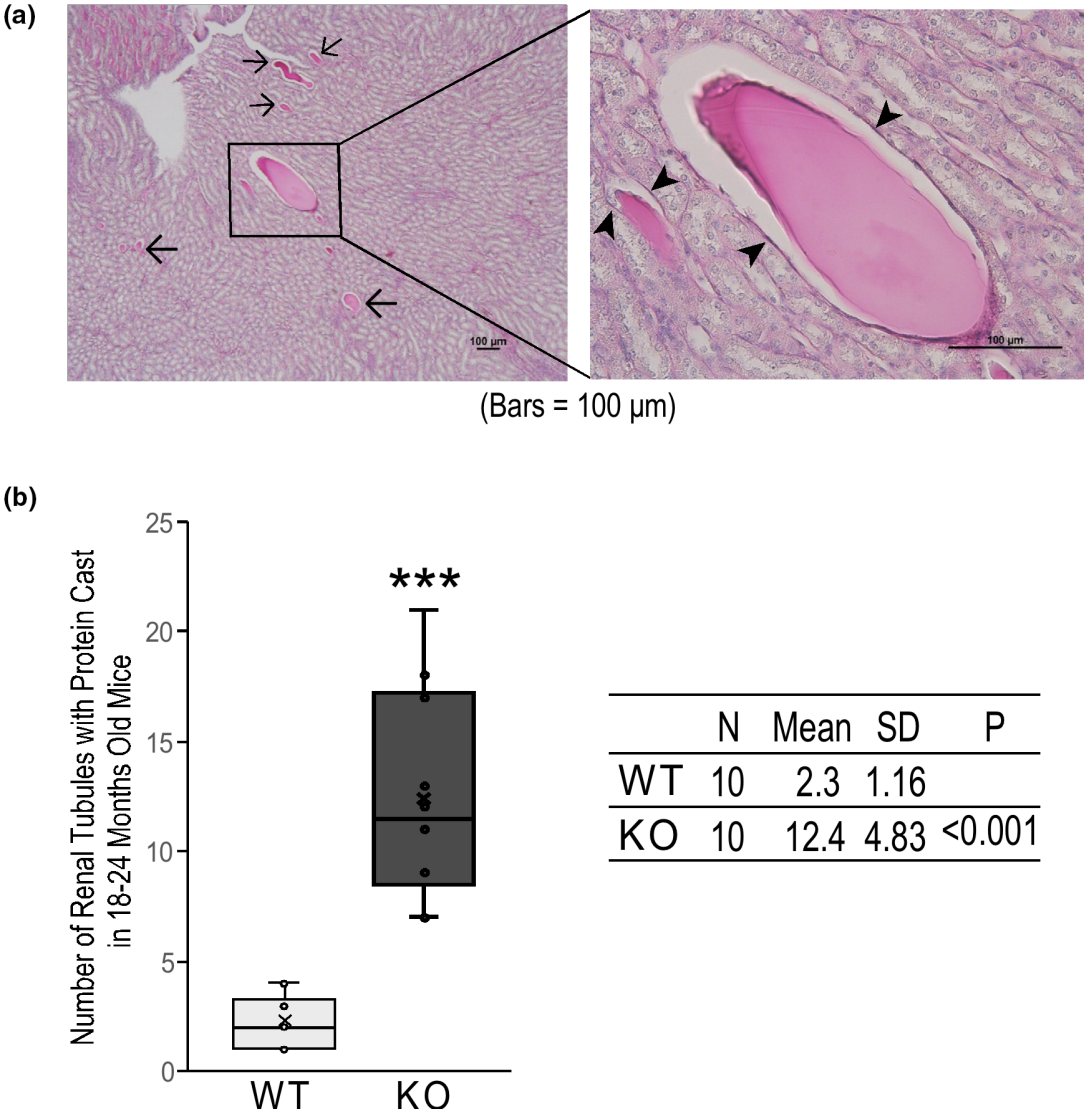
Aging calponin 2 KO mice had significantly higher number of dilated renal tubules containing proteinaceous casts. (a) The PAS stained images demonstrate the dilated renal tubules containing proteinaceous casts. The blow‐up panel to the right demonstrates that most of the cells lining the dilated tubules were atrophied. The arrowheads indicate the nucleus of the atrophic tubular epithelium. (b) The box and whisker plots and data table demonstrate that aging calponin 2 KO mice had significantly higher number of dilated renal tubules containing proteinaceous casts as compared to that of age‐matched WT mice, indicating severe proteinuria developed in aging calponin 2 KO mice. Values are means ± SD. ***p < 0.001 compared with WT control in two‐tail Student’s *t* test.

Figure 3a shows a representative PAS‐stained mid‐sagittal section of the kidney from an aging calponin 2 KO mouse to demonstrate the proteinaceous casts in dilated renal tubules with various degree of cyst formation due to protein leaking from the glomeruli. The dilated renal tubules with protein casts were mostly found in the inner strip of the outer medulla of the kidney (Figure 1a). The quantitative data in Figure 3b show significantly higher number of dilated renal tubules containing proteinaceous casts in calponin 2 KO mouse kidney than that of age matched WT controls, confirming the severe proteinuria developed in aging calponin 2 KO mice.

### 3.2 Calponin 2 KO mice showed age‐progressive high percentage of glomerulus with low G/B ratio

**FIGURE 4:**
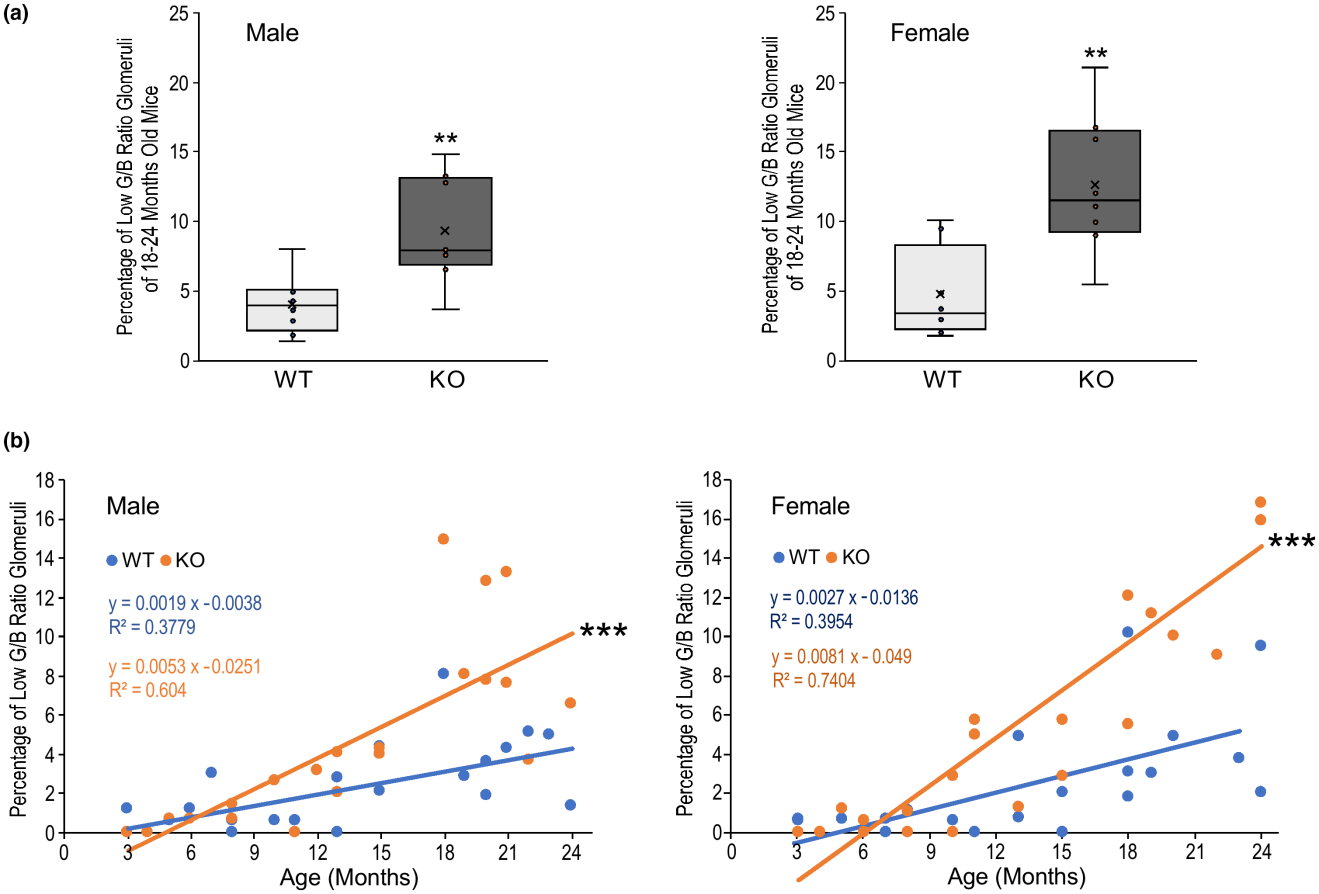
Calponin 2 KO mice age‐progressively develop significantly higher percentage of low G/B ratio glomeruli. (a) The box and whisker plot comparison of the percentage of glomeruli with low G/B ratio shows that at 18 to 24 months old, both male and female calponin 2 KO mice showed significantly higher percentages of glomeruli with low G/B ratio than that of age‐ and sex‐matched WT control. (**p < 0.01). (b) The correlation plots show while the percentages of glomeruli with low G/B ration were correlated with age in both male and female WT mice reflecting physiological age‐related degenerations, steeper positive correlations of the percentage of glomeruli with low G/B ratio with age were found in male and female calponin 2 KO mice as analyzed by linear regression and Pearson's correlation and ANOVA test for statistical significance (***p < 0.001), demonstrating significantly accelerated degeneration of glomeruli during aging. Values are means ± SD.

The density of glomerulus was not significantly different between calponin KO and WT mouse kidneys at both young adult and old ages examined (Table 2). However, aging calponin 2 KO mice showed a significantly higher percentage of glomeruli with low G/B ratio than that of age‐matched WT control while no statistically significant difference was observed at the young adult age (Table 2). The significantly higher percentage of glomeruli with low G/B ratio in 18–24 months old calponin 2 KO mice versus that of WT controls is shown in Figure 4a. It is worth noting that most of the glomeruli in aging calponin 2 KO mouse kidneys showed similar morphology to that of WT control with ~13% having low G/B ratio. While obvious lesion only occurred in this relatively small proportion of glomeruli and did not cause signs of clinical renal failure, severe proteinuria has developed as an early sign of renal disease.

**TABLE 2:** Calponin 2 KO mice exhibited age‐progressive increases in the percentage of glomerulus with low glomerular/Bowman's capsule ratio. The data demonstrate that total glomerular counts of calponin 2 KO and WT groups did not show significant differences at all three age groups of mouse in both sexes. Comparing the 18–24 months old groups, both male and female calponin 2 KO mice showed significantly higher percentage of glomerulus with low G/B ratio than that of the WT counterparts. *p* Values are listed to indicate statistical significance between the KO and WT groups in two‐tailed Student’s *t* test. Values are means ± SD

Age	Male group 1 (3–8 M)		Male group 2 (10–15 M)		Male group 3 (18–24 M)		Female group 1 (3–8 M)		Female group 2 (10–15 M)		Female group 3 (18–24 M)	
Strain (*n*)	WT (7)	KO (7)	WT (7)	KO (7)	WT (8)	KO (8)	WT (7)	KO (7)	WT (7)	KO (7)	WT (8)	KO (8)
TG	165.7 ± 9.4	157.0 ± 12.4	152.1 ± 11.7	158.3 ± 12.2	153.6 ± 10.9	153.8 ± 11.4	160.3 ± 12.9	166.7 ± 10.2	154.9 ± 12.0	155.3 ± 15.5	161.5 ± 8.4	156.8 ± 19.9
Low G/B	1.6 ± 1.7	0.7 ± 0.8	2.1 ± 2.3	7.3 ± 4.5	6.0 ± 3.7	14.6 ± 6.7	0.9 ± 0.7	5.7 ± 2.1	1.7 ± 2.6	12.1 ± 2.2	7.6 ± 5.1	21.4 ± 7.0
Low G/B(%)	0.94 ± 1.03	0.48 ± 0.52	1.49 ± 1.64	2.88 ± 1.51	4.01 ± 2.12	9.33 ± 3.87	0.54 ± 0.41	0.42 ± 0.55	1.16 ± 1.78	3.33 ± 2.22	4.76 ± 3.27	12.69 ± 4.99
*p* value	0.305		0.125		0.004		0.629		0.067		0.002	

The course of age‐progressive renal glomerular degeneration in calponin 2 KO mice was compared with that of WT controls. The results of correlation analysis in Figure 4b demonstrated that the loss of calponin 2 significantly accelerates the degeneration of glomeruli as shown by the faster rate of age‐progressive increase of the percentage of glomeruli with low G/B ratio.

### 3.3 Calponin 2 expresses at high levels in Podocytes

**FIGURE 5:**
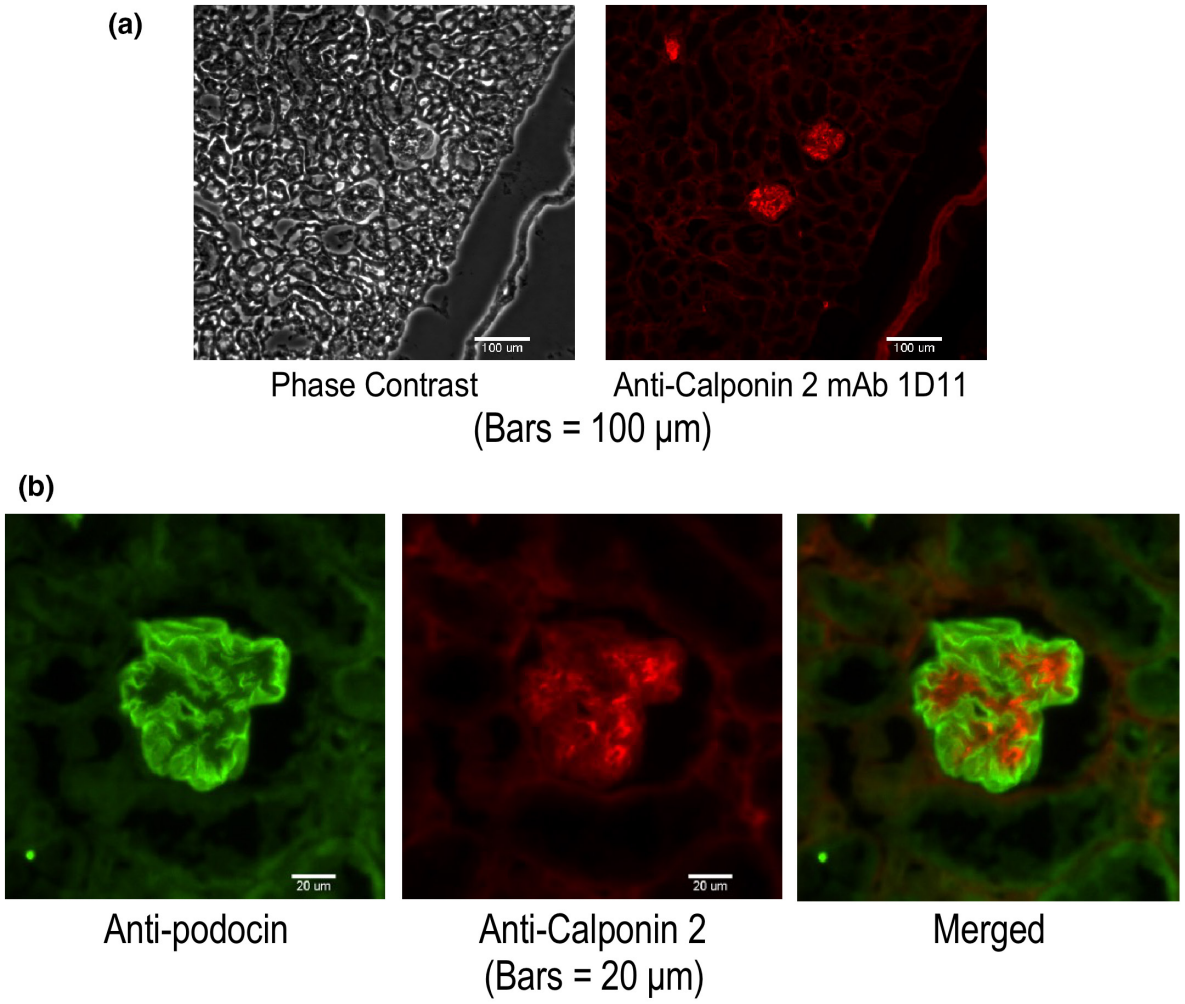
Expression of calponin 2 in renal glomerulus. (a) Frozen section of a WT mouse kidney stained with anti‐calponin 2 mAb 1D11 detected the expression of calponin 2 in glomerulus. (b) Immunofluorescent images of frozen sections of young adult WT mouse kidney with mAb 1D11 (red) and anti‐podocin antibody (green) showed calponin 2 and podocin expressions in the glomerular tufts. Podocin expressed linearly in the peripheral edge along the capillary tufts which represent the foot processes of podocytes whereas calponin 2 expressed in the central portion of the capillary loop implying the cell body of podocytes.

Frozen sections of WT mouse kidney stained with anti‐calponin 2 mAb 1D11 detected calponin 2 in the glomerular tufts. While podocin expressed linearly in the peripheral edge along the capillary tufts which represent foot processes of the podocyte, calponin 2 was detected in the central portion of the capillary loop corresponding to the bodies of podocytes (Figure 5a). At higher magnification, the expression of podocin in glomeruli of aging WT mice showed continuously lining the vessel tufts (Figure 5b).

**FIGURE 6:**
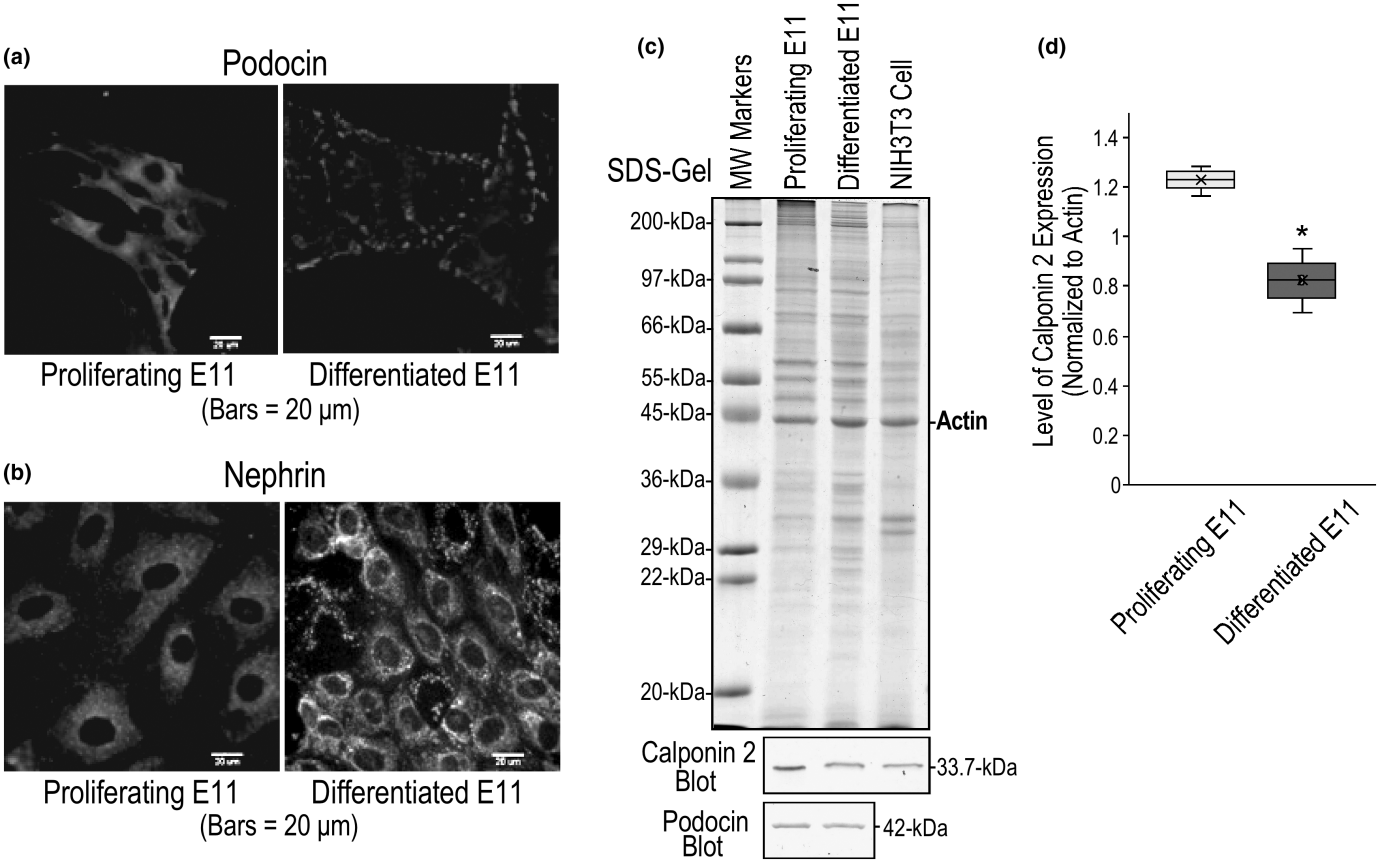
High level expression of calponin 2 in podocyte. (a) Immunofluorescence staining of adherent E11 cells cultured on collagen coated cover slip with anti‐podocin antibody showed the expression of podocin. In proliferating cells (left image), podocin shows diffused distribution and more concentrated around the nucleus. In 14‐day differentiated podocytes (right image), podocin forms clusters along the edges of the cells. (b) Immunofluorescence microscopic imaging of proliferating (left image) and differentiated (right image) E11 cells also detected high level expression of nephrin, further demonstrating the podocyte feature of this cell line. Nephrin showed diffused distribution in proliferating E11 cells with higher concentration around the nucleus and formed clusters after differentiation in culture for 14 days. (c) Western blot analysis of total lysate of E11 podocytes showed the expression of calponin 2 in both proliferating and differentiated cultures. Whereas as no significant difference in the expression levels of podocin relative to actin was found between proliferative and differentiated states, calponin 2 is expressed at a significantly lower level in differentiated E11 cells, which is quantified by densitometry analysis of the Western blots normalized to the level of actin in parallel SDS‐gels (d). The quantification was from four repeated experiments under the same culture conditions (*n* = 4). *p < 0.05 in two‐tailed Student’s *t* test. Values are means ± SD.

Adherent proliferating mouse podocyte cell line E11 cultured on collagen coated cover slips showed podocin expression with a diffused distribution with higher concentration around the nucleus. After 14 days of differentiation, podocin forms clusters along the edges of the cells (Figure 6a). Immunofluorescence microscopy also detected the expression of nephrin in proliferating and differentiated E11 podocytes (Figure 6b). The data confirmed the characteristics of podocytes of this cell line for use in the studies of calponin in renal podocytes.

Western blot analysis of total protein extracts of E11 podocytes detected high levels of calponin 2 in proliferating state and after 14 days of differentiation in culture (Figure 6c). Normalized to the level of actin, proliferating podocytes showed higher expression of calponin 2 than that of differentiated podocytes while no difference was found in the levels of podocin (Figure 6d).

**FIGURE 7:**
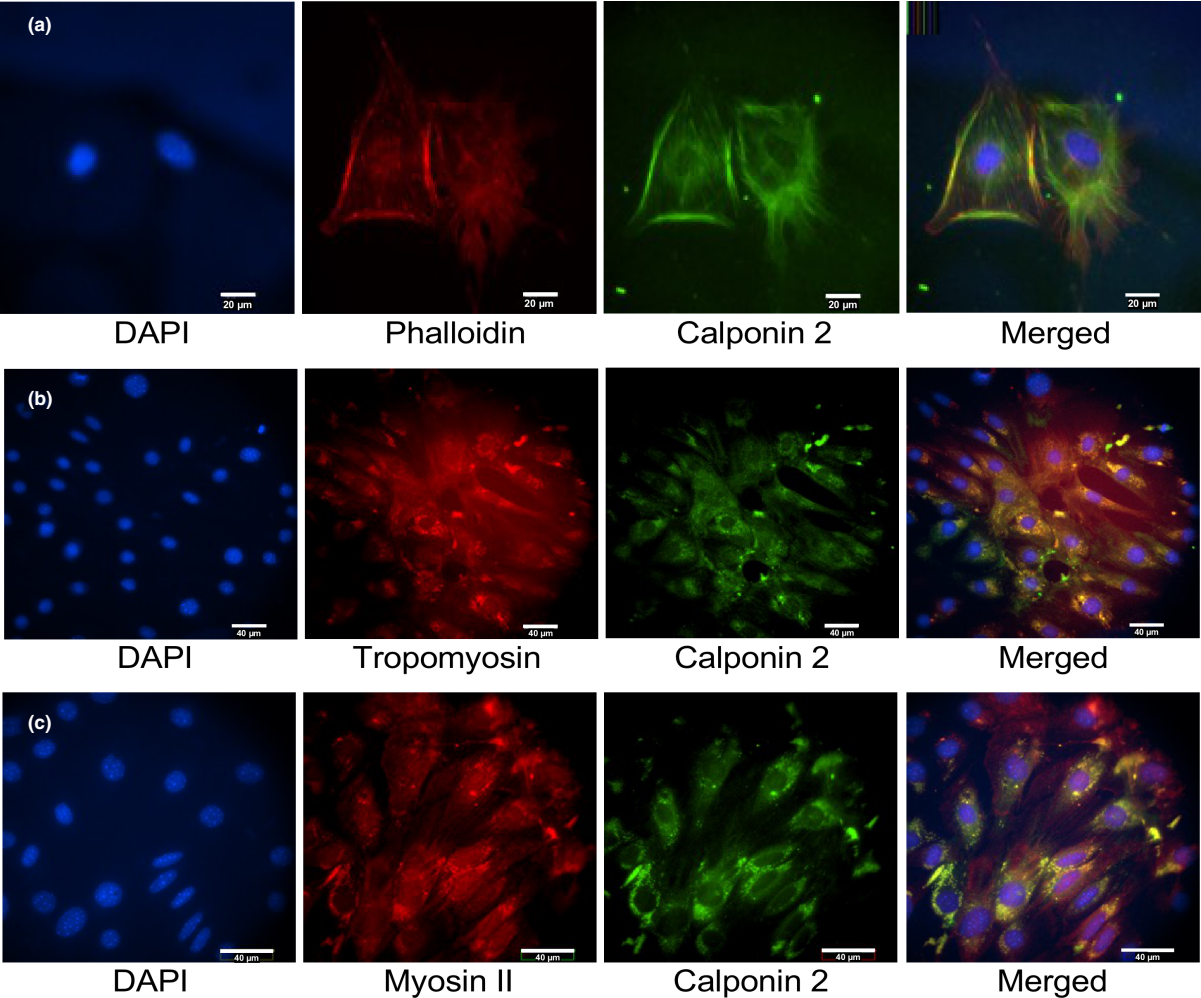
Calponin 2 colocalize with actin microfilaments, tropomyosin and myosin IIA in podocytes. Double immunofluorescent staining of E11 podocytes differentiated in culture with anti‐calponin 2 mAb 1D11 and phalloidin or anti‐tropomyosin and anti‐myosin IIA antibodies demonstrate the colocalization of calponin 2 with F‐actin microfilament (a), tropomyosin (b) and myosin IIA (c). DAPI counter stain was used to show the location of nucleus.

Immunofluorescence microscopic studies further showed that calponin 2 colocalizes with actin microfilaments, tropomyosin, and myosin IIA in E11 podocytes differentiated in culture for 14 days (Figure 7). The results indicate its association with the actin cytoskeleton of podocytes with extensions into the cellular projections (Figure 7a). The colocalization of calponin 2 with myosin IIA (Figure 7c) indicates its function in regulating myosin motor activity and mechanical tension equilibrium (Hossain et al., 2016) in the cytoskeleton of podocytes.

### 3.4 Calponin 2 KO mice developed effacement of the podocyte foot processes and increased thickness of the glomerular basement membrane

**FIGURE 8:**
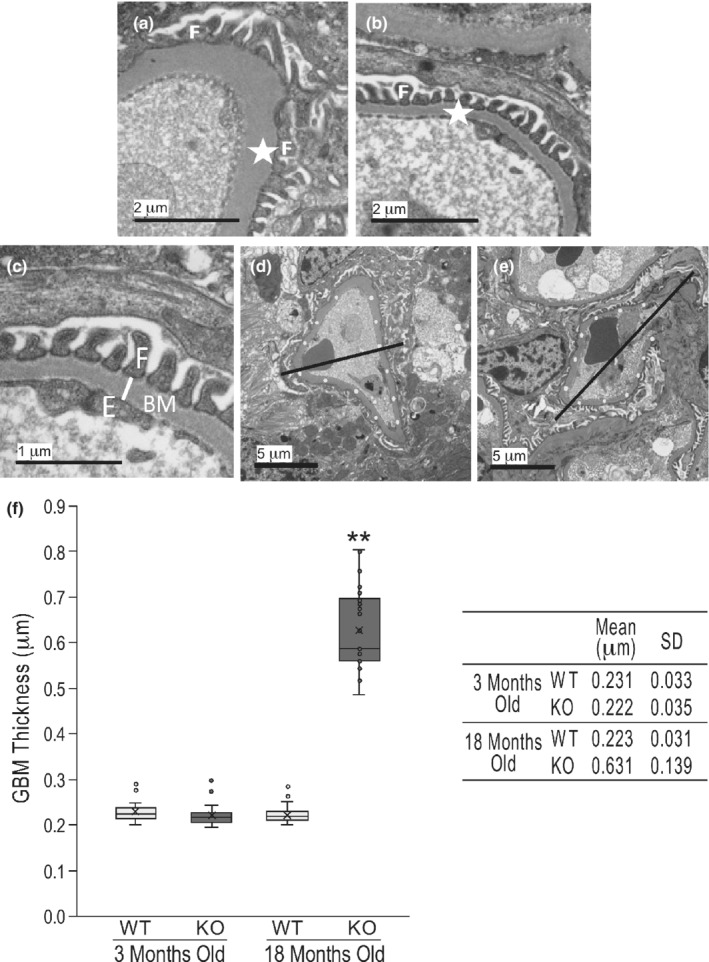
Increased thickness of glomerular basement membrane in aging calponin 2 KO mice. EM imaging showed that (a) effacement of the podocyte foot processes in 18 months old calponin 2 KO mice (the white F indicates two effaced foot processes and the white star marks the location of GBM, the black bar represents 2 μm), (b) age‐matched WT mouse with normal interdigitated foot processes (indicated by the white F), (c) on a section of 18 months old WT mouse kidney, GBM thickness measured from the lower tip of the foot process of podocyte (indicated by the white F) to the upper end of the endothelium (indicated by the white E) perpendicular to the long axis of the GBM as indicated by the white line, and (d & e) on sections of a 18 months old calponin 2 KO mouse kidney, average GBM thickness calculated by dividing the capillary loop into two halves, measuring random points at equal distance (the white dots) and avoiding the tip of a sharp angle or loop joining mesangial cells without coverage by podocyte. (f) Quantitative data in the box and whisker plots and inset table show the significantly increased GBM thickness in aging calponin KO mice as compared to that of WT controls and young calponin KO mice. Values are means ± SD. **p < 0.01 in two‐tailed Student’s *t* test.

EM imaging showed that the GBM was significantly thicker in aging calponin 2 KO mouse kidneys as compared to that of age‐matched WT controls whereas no difference was found between young adult KO and WT mice (Figure 8). Effacement of the podocyte foot processes was found in aging but not young adult KO mice or WT controls (Figure 8). The ultrastructural data provide evidence that the loss of calponin 2 results abnormality in podocyte structure and function to cause age‐progressive glomerular degeneration and proteinuria.

## 4 DISCUSSION

Calponin 2 is found in a wide range of cell types originated from all three germ layers. Under normal physiological conditions, diverse types of calponin 2 expressing cells including cells that are under high mechanical tension, such as lung alveolar cells and kidney podocytes, or cells with high proliferation rates, or actively migrating cells (Feng et al., 2019). The expression of calponin 2 is positively regulated by mechanical tension in the cytoskeleton (Hossain et al., 2005; Hossain et al., 2006). The cell type specific expressions of calponin 2 is consistent with its role in maintaining cytoskeleton stability against mechanical tension environment. Our present study provides novel evidence that calponin 2 plays a critical role in the structural and functional integrity of renal podocytes that are adapted to a unique mechanical environment due to the physiologically high hydrostatic filtration pressure in renal glomeruli. Animals naturally lack calponin 2, such as rabbits, have been reported (Plazyo et al., 2020). Deletion of *CNN2* gene in human has also been reported (Silversides et al., 2012). Calponin 2 KO mice have a life expectancy equivalent to that of the wild type littermates, providing an informative model for the study of chronic diseases. Our finding that calponin 2 KO mice develop age‐progressive proteinuria and podocyte injury leads to the following observations.

### 4.1 High level expression and actin cytoskeleton‐association of calponin 2 in podocytes

Our data show that calponin 2 is expressed at high levels in podocytes, as evident in a podocyte cell line. Immunofluorescence imaging demonstrated the colocalization of calponin 2 and actin stress fibers. In vitro differentiated podocytes showed peripheral localization of podocin which plays a role in connecting neighboring podocyte to slit diaphragm. However, no colocalization of calponin 2 and podocin was found. For calponin's primary activity as a regulator of myosin motors, its centralized localization in podocytes may indicate functions in maintaining the myosin motor‐based cellular tension (Hossain et al., 2016) that determines cell adhesion and connection with neighboring cells.

### 4.2 Deletion of calponin 2 results in age‐progressive proteinuria

While calponin 2 KO mice have a similar life expectancy as that of the WT mice, the age‐progressive proteinuria found in the present study indicates chronic deterioration of kidney function. Previous research showed that decaying renal function in aging C57BL/6 strain of mice is less likely to produce proteinuria in comparison with other strains (Hackbarth & Harrison, 1982; Ma & Fogo, 2003), which was confirmed in the WT control mice in our present study. In addition, many nephrotoxic approaches to producing proteinuria are genetic background dependent and ineffective in C57BL/6 mice (Ishola Jr. et al., 2006). Our calponin 2 KO mice are in C57BL/6 background and, therefore, the development of proteinuria at advanced age provides convincing evidence for the critical role of calponin 2 in maintaining the structural integrity and function of renal glomeruli. This finding suggests that calponin 2 regulation of the tension adaptation of actin cytoskeleton stability is a pivotal point in the pathogenesis of proteinuria. The loss of calponin 2 function results in progressive degeneration and protein leakage of glomeruli presumably by causing mechanical tension‐related podocyte injuries. Our finding in the mouse model urges clinical consideration of including *CNN2* genetic testing for etiologically unknown proteinuria patients.

### 4.3 Calponin 2 as a novel molecular target in podocytopathy

Age related loss of podocytes has been reported in rodents (Bitzer & Wiggins, 2016; Hodgin et al., 2015). However, the mechanism of podocyte loss in aging kidney remains unclear. Our EM results show increased thickness of GBM in aging calponin 2 KO mice. Thicker GBM is a known manifestation of kidney diseases, such as diabetic nephropathy and minimal change disease (MCD). There is strong evidence for podocyte dysfunction with loss of counterbalancing force against high hydrostatic pressure in the glomerular capillaries to cause increased GBM thickness (Butt et al., 2020). Increased GBM thickness increases the size of the filtration pores, lowering the ability of retaining albumin and other plasma macromolecules to cause proteinuria. Podocytopathies, such as that occurring in MCD, focal segmental glomerular sclerosis (FSGS) and pre‐eclampsia, share similar morphological changes in the glomerulus, including effacement of podocyte foot processes and increased GBM thickness (Craici et al., 2014). These podocytopathies develop gradually with varying degrees of proteinuria, similar to the phenotype of calponin 2 KO mice. Detachment of podocytes from the glomerular basement membrane caused by excess mechanical tension or impaired adhesion has been postulated as a mechanism in podocyte losses (Kriz & Lemley, 2015).

Our previous studies detected calponin 2 in total protein extracts of multiple organs, including the kidney (Hossain et al., 2006). However, the result does not indicate the expression of calponin 2 in podocytes that only contributes to a small fraction of the kidney mass of which renal tubules, collecting tubules, blood vessels, and connective tissues are major components with calponin 2 expressing cells. Current methods to isolate glomeruli are impractical to be used for quantification of calponin 2 in the podocytes because the glomerulus is composed of other types of cells (e.g., vascular endothelial, smooth muscle cells and fibroblasts that express calponin 2 (Hossain et al., 2006; Qian et al., 2021; Tang et al., 2006). There is no reported method to purify podocytes in sufficient number for reliable quantification of calponin 2 expression. Aging organs including the kidney often experience chronic fibrosis (Karam & Tuazon, 2013). Since fibroblasts and inflammatory cells express high levels of calponin 2 (Hsieh et al., 2022; Huang et al., 2008), examinations of organ level expression of calponin 2 would not be effective for detecting aging‐related decrease in calponin 2.

Nonetheless, it is important to understand whether aging‐related decreases of calponin 2 in podocytes are a cause of proteinuria in human patients. Follow‐up studies are required to investigate calponin 2 regulation in podocytes during aging for significance in the pathogenesis of proteinuria.

### 4.4 The role of calponin 2 in cytoskeleton dynamics and stability of podocytes

Cytoskeleton network in healthy podocytes contain noncontractile actin microfilaments in the foot processes, which are connected to the contractile actin cables in the major processes and cell body (Suleiman et al., 2017). Podocyte injury leads to the disassembly and reorganization of these interconnected structure of actin framework (Suleiman et al., 2017). Calponin 2 is known to function in stabilizing noncontractile actin cytoskeleton (Panasenko & Gusev, 2001), which exists in podocyte foot processes, and to inhibit actomyosin ATPase in the contractile actin cables (Taniguchi, 2005), which locate in the major processes and cell body. Myosin IIA has been shown to play critical roles in podocyte actin organization, contraction, and attenuation of cell motility (Bondzie et al., 2016). Therefore, our data indicate a role of calponin 2 in the structural stability and function of podocytes. A loss or abnormality of calponin 2 would result in dysregulated myosin motor activity to increase the dynamics of actin cytoskeleton and decrease the structural stability of podocytes with decreased resistance against hydrostatic filtration‐generated mechanical tension. Our findings in the present study support that the loss of calponin 2 regulatory function in podocytes destabilizes the actin cytoskeleton, lowers the durability of podocyte foot processes, leads to the increased GBM thickness and causes proteinuria. The hypothesis that calponin 2 may be a molecular target in the pathogenesis of podocyte injury and proteinuria is worth further investigating toward the underlying mechanisms for therapeutical development.

## AUTHOR CONTRIBUTIONS

Jian‐Ping Jin conceived and designed the research; Tzu‐Bou Hsieh designed and conducted the experiments and analyzed data; Tzu‐Bou Hsieh prepared the figures and drafted the manuscript; Tzu‐Bou Hsieh and Jian‐Ping Jin revised figures, tables and text and approval the final version of the manuscript.

## FUNDING INFORMATION

This study was supported in part by a grant from the National Institutes of Health (HL138007) to Jian‐Ping Jin. Tzu‐Bou Hsieh was a recipient of Women's Reproductive Health Research fellowship program from the National Institute of Child Health and Human Development (HD001254 to Dr. Chaur‐Dong Hsu).

## CONFLICT OF INTEREST

The authors declare that they have no conflict of interest.
